# Hyperoxia Is Not Associated With 30-day Survival in Out-of-Hospital Cardiac Arrest Patients Who Undergo Extracorporeal Cardiopulmonary Resuscitation

**DOI:** 10.3389/fmed.2022.867602

**Published:** 2022-05-09

**Authors:** Mioko Kobayashi, Masahiro Kashiura, Hideto Yasuda, Kazuhiro Sugiyama, Yuichi Hamabe, Takashi Moriya

**Affiliations:** ^1^Tertiary Emergency Medical Center, Tokyo Metropolitan Bokutoh Hospital, Tokyo, Japan; ^2^Department of Emergency and Critical Care Medicine, Saitama Medical Center, Jichi Medical University, Saitama, Japan

**Keywords:** blood gas analysis, cardiopulmonary resuscitation, extracorporeal membrane oxygenation, hyperoxia, post-cardiac arrest syndrome, cardiac arrest

## Abstract

**Introduction:**

The appropriate arterial partial pressure of oxygen (PaO_2_) in patients undergoing extracorporeal cardiopulmonary resuscitation (ECPR) for out-of-hospital cardiac arrest (OHCA) remains unclear. The present study aimed to investigate the relationship between hyperoxia and 30-day survival in patients who underwent ECPR.

**Materials and Methods:**

This single-center retrospective cohort study was conducted between January 2010 and December 2018. OHCA patients who underwent ECPR were included in the study. Exclusion criteria were (1) age <18 years, (2) death within 24 h after admission, (3) return of spontaneous circulation at hospital arrival, and (4) hypoxia (PaO_2_ < 60 mmHg) 24 h after admission. Based on PaO_2_ at 24 h after admission, patients were classified into normoxia (60 mmHg ≤ PaO_2_ ≤ 100 mmHg), mild hyperoxia (100 mmHg < PaO_2_ ≤ 200 mmHg), and severe hyperoxia (PaO_2_ > 200 mmHg) groups. The primary outcome was 30-day survival after cardiac arrest, while the secondary outcome was 30-day favorable neurological outcome. Multivariate logistic regression analysis for 30-day survival or 30-day favorable neurological outcome was performed using multiple propensity scores as explanatory variables. To estimate the multiple propensity score, we fitted a multinomial logistic regression model using the patients' demographic, pre-hospital, and in-hospital characteristics.

**Results:**

Of the patients who underwent ECPR in the study center, 110 were eligible for the study. The normoxia group included 29 cases, mild hyperoxia group included 46 cases, and severe hyperoxia group included 35 cases. Mild hyperoxia was not significantly associated with survival, compared with normoxia as the reference (adjusted odds ratio, 1.06; 95% confidence interval: 0.30–3.68; *p* = 0.93). Severe hyperoxia was also not significantly associated with survival compared to normoxia (adjusted odds ratio, 1.05; 95% confidence interval: 0.27–4.12; *p* = 0.94). Furthermore, no association was observed between oxygenation and 30-day favorable neurological outcomes.

**Conclusions:**

There was no significant association between hyperoxia at 24 h after admission and 30-day survival in OHCA patients who underwent ECPR.

## Introduction

Out-of-hospital cardiac arrest (OHCA) remains a major health burden worldwide (1). The American Heart Association reports that at the time of hospital discharge, the survival rate of patients with OHCA is approximately 10%, which remains low despite advances in cardiopulmonary resuscitation (CPR) and post-cardiac arrest syndrome care (2).

Extracorporeal cardiopulmonary resuscitation (ECPR) is the administration of veno-arterial extracorporeal membrane oxygenation (ECMO) to cardiac arrest patients who are refractory to conventional CPR (3). The main purpose of ECPR is to restore circulation and gas exchange, and it has been shown to improve clinical outcomes after OHCA (4–6). ECMO provides time for interventions that are necessary for achieving adequate spontaneous circulation, including percutaneous coronary intervention, pulmonary thrombectomy, and rewarming.

Some studies have reported that hyperoxia contributes to the deterioration of patients with post-cardiac arrest syndrome (7). Therefore, the latest guidelines for cardiac arrest management recommend avoiding hyperoxia after the return of spontaneous circulation (8, 9). During ECPR, the sweep gas provides supraphysiological levels of oxygenation, which can exacerbate post-cardiac arrest syndrome. However, clinical studies evaluating hyperoxia associated with ECPR are few (10–12). Furthermore, in previous studies, the site of blood sample collection for blood gas analysis was not specified (10–12). Therefore, the influence of hyperoxia on neurological outcome and mortality in patients who undergo ECPR for OHCA remains unclear.

The purpose of this study was to investigate the relationship between oxygenation and survival and favorable neurological outcome in adult patients who underwent ECPR.

## Materials and Methods

### Study Design and Setting

This retrospective study was conducted in a tertiary emergency center that serves a population of approximately 1,800,000 in the eastern Tokyo metropolitan area of Japan. The institutional review board of Tokyo Metropolitan Bokutoh Hospital approved the study. The requirement for informed consent was waived because of the observational study design that posed minimal risk to patients and preserved their anonymity. An opportunity to opt out from the registry was provided for patients and their respective families.

### Participants

Adult OHCA patients (≥ 18 years old) who underwent ECPR between January 2010 and December 2018 were included in the study. The following were the exclusion criteria: (i) age <18 years, (ii) death within 24 h after admission, (iii) restoration of spontaneous circulation upon hospital arrival, and (iv) hypoxia (partial pressure of arterial oxygen [PaO_2_] <60 mmHg) 24 h after admission.

The indications for ECPR at the study center were one of the following: (i) initial shockable rhythm, time from arrest to hospital arrival <30 min, witness of cardiac arrest by a bystander, and age <65 years; or (ii) witness of cardiac arrest by emergency medical service personnel, presumed reversible etiology (e.g., cardiac disease, pulmonary embolism, incidental hypothermia, and drug overdose), and age <70 years (13). Outside these circumstances, ECPR was performed at the discretion of the emergency physician. ECPR was performed in the emergency room immediately after hospital arrival. Both the outflow and inflow cannulas were inserted percutaneously into contralateral or ipsilateral femoral vessels by emergency physicians. For the femoral artery, 15/16 Fr cannulas were used; for the femoral vein, 21/22 Fr cannulas were used. The blood circuit set, including a pump and membrane oxygenator, was primed using normal saline with 3,000 units of heparin. The ECMO pump flow rate was set between 3 and 4 L/min at the discretion of the emergency physician. After the ECMO pump was turned on, an arterial line was inserted into the right radial or brachial artery, and it reflected cerebral oxygenation.

### Data Collection

Data (patients' demographics, cardiac arrest characteristics, treatment, laboratory data, and outcomes) were retrieved from the electronic medical records. The following patients' demographics and pre-hospital data were collected: age, sex, witness status (emergency medical service personnel or others), bystander CPR, etiology of cardiac arrest (cardiac or non-cardiac), initial cardiac rhythm, pre-hospital adrenaline administration, and pre-hospital shock delivery. In addition, the following in-hospital factors and outcomes were retrieved: time from arrest to ECMO pump-on, blood gas analysis data (pH, PaO_2_, partial pressure of arterial carbon dioxide [PaCO_2_], bicarbonate ion concentration [${\text{HCO}}_{3}^{-}$], and lactate level), intra-aortic balloon pump use, percutaneous coronary intervention, and cerebral performance category 30 days after cardiac arrest. Every 4 h, at most, blood samples for gas analysis were obtained from an arterial line inserted into the right radial artery or brachial artery.

### Exposure and Definition

The eligible patients were divided into the following three groups according to their PaO_2_ levels 24 h after admission: normoxia group, 60 mmHg ≤ PaO_2_ ≤ 100 mmHg; mild hyperoxia group, 100 mmHg < PaO_2_ ≤ 200 mmHg; and severe hyperoxia group, 200 mmHg < PaO_2_ (14).

### Outcome Measures

The primary outcome was 30-day survival after cardiac arrest. The secondary outcome was 30-day favorable neurological outcome after cardiac arrest. A favorable neurological outcome was defined as cerebral performance category 1 or 2. The possible cerebral performance categories were 1) good cerebral recovery, 2) moderate cerebral disability, 3) severe cerebral disability, 4) coma or vegetative state, and 5) death or brain death (15).

### Statistical Analyses

Continuous variables are presented as medians and interquartile ranges, while categorical variables are presented as counts and percentages. Continuous variables were analyzed with the Kruskal-Wallis test, and categorical variables in the three groups were analyzed with a Fisher's exact test.

We used multiple propensity score analysis to adjust and control for multiple confounding factors in the comparison of the three groups (16). A multiple propensity score is the conditional probability of a patient being categorized into one of three or more groups, given baseline covariates. First, we created a multinomial logistic regression model by setting one of the three PaO_2_ groups as the dependent variable. The following covariates were used to calculate the multiple propensity scores: age, sex, type of witness, bystander administration of CPR, initial cardiac rhythm, pre-hospital shock delivery, pre-hospital adrenaline administration, etiology, percutaneous coronary intervention, intra-aortic balloon pump use, and time from arrest to ECMO pump-on. Second, we performed binomial logistic regression analysis to determine the adjusted odds ratios (ORs) of the PaO_2_ level group for 30-day survival or 30-day favorable neurological outcome after cardiac arrest, adjusting for multiple propensity scores and variables at 24 h after admission, including PaCO_2_, pH, ${\text{HCO}}_{3}^{-}$, lactate level, and mean blood pressure.

In addition, we performed sensitivity analysis using a similar multiple propensity score, grouping patients by PaO_2_ 12 h after admission.

ORs and 95% confidence intervals (CIs) were calculated. All statistical tests were two-sided, and *p* values <0.05 were considered significant. All statistical analyses were conducted using Statistical Package for the Social Sciences version 26.0 for Mac (IBM Corp., Armonk, NY, USA).

## Results

### Patient Enrollment

**Figure 1:**
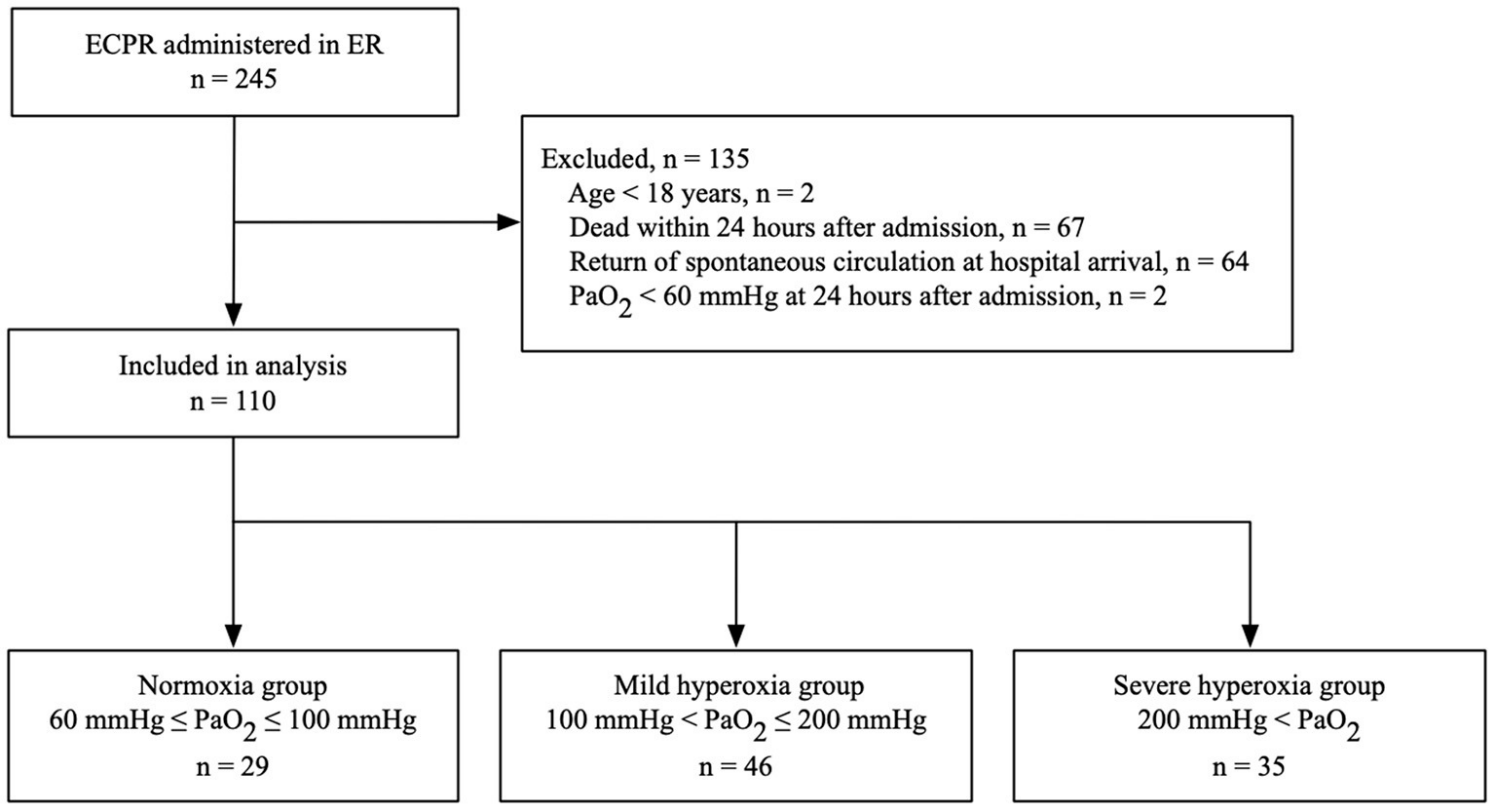
Flowchart of study patients who underwent ECPR. ECPR, extracorporeal cardiopulmonary resuscitation; ER, emergency room; PaO_2_, arterial partial pressure of oxygen.

Between January 2010 and December 2018, 245 patients underwent ECPR in the emergency room of the study center. After excluding 2 patients under 18 years, 67 patients who died within 24 h after admission, 64 patients with return of spontaneous circulation at hospital arrival, and 2 patients with PaO_2_ < 60 mmHg at 24 h after admission, 110 patients were finally included in the study (Figure 1).

### Patients' Characteristics and Outcomes

Demographic and pre-hospital characteristics of the patients are shown in Table 1. The median age was 59 years (interquartile range, 45–64 years), and 87.3% of the patients were men. Witness status and pre-hospital adrenaline administration were significantly different among groups. In-hospital variables, including blood gas data and clinical outcomes, are shown in Table 2. The median PaO_2_ level 24 h after admission was 145 mmHg (interquartile range, 99–237 mmHg) in all patients. A total of 29, 46, and 35 patients were categorized into the normoxia, mild hyperoxia, and severe hyperoxia groups, respectively (Figure 1). The pH, ${\text{HCO}}_{3}^{-}$, lactate level, and mean arterial blood pressure 24 h after admission were significantly different among groups. There was no statistically significant difference in 30-day survival or 30-day favorable neurological outcome among the three groups.

**Table 1:** Demographic and pre-hospital characteristics of the study population.

**Factor**	**Overall** **(*n* = 110)**	**Normoxia** **(*n* = 29)**	**Mild hyperoxia** **(*n* = 46)**	**Severe hyperoxia** **(*n* = 35)**	***p*-value**
Age, years	59 [45–64]	61 [46–64]	56 [45–65]	53 [45–62]	0.27
Male	96 (87.3)	25 (86.2)	39 (84.8)	32 (91.4)	0.71
Witness of cardiac arrest					0.042
by EMS personnel	41 (37.3)	11 (37.9)	23 (50.0)	7 (20.0)	
by others	57 (51.8)	14 (48.3)	21 (45.7)	22 (62.9)	
Bystander CPR	74 (67.3)	19 (65.5)	36 (78.3)	19 (54.3)	0.072
Cardiac origin	89 (80.9)	25 (86.2)	33 (71.7)	31 (88.6)	0.14
Initial cardiac rhythm monitored					0.25
VF	68 (61.8)	19 (65.5)	23 (50.0)	26 (74.3)	
Pulseless VT	5 (4.5)	1 (3.4)	4 (8.7)	0 (0.0)	
PEA	29 (26.4)	6 (20.7)	15 (32.6)	8 (22.9)	
Asystole	8 (7.3)	3 (10.3)	4 (8.7)	1 (2.9)	
Prehospital adrenaline administration	92 (83.6)	20 (69.0)	43 (93.5)	29 (82.9)	0.021
Prehospital shock delivery	79 (71.8)	23 (79.3)	28 (60.9)	28 (80.0)	0.11

*Continuous variables are presented as median [interquartile range]. Categorical variables are presented as count (percentage)*.

*CPR, cardiopulmonary resuscitation; EMS, emergency medical service; PEA, pulseless electrical activity; VF, ventricular fibrillation; VT, ventricular tachycardia*.

**Table 2:** In-hospital variables and clinical outcomes of the study population.

**Factor**	**Overall** **(n = 110)**	**Normoxia** **(n = 29)**	**Mild hyperoxia** **(n = 46)**	**Severe hyperoxia** **(n = 35)**	***p*-value**
Time from arrest to ECMO pump-on, min	40 [31–49]	36 [23–47]	39 [31–47]	44 [37–52]	0.085
Percutaneous coronary intervention	45 (40.9)	16 (55.2)	13 (28.3)	16 (45.7)	0.054
Intra-aortic balloon pump	79 (71.8)	20 (69.0)	32 (69.6)	27 (77.1)	0.77
PaO_2_ 12 h after admission, mmHg	217 [141–317]	172 [100–263]	190 [130–273]	326 [258–457]	<0.001
PaCO_2_ 12 h after admission, mmHg	35 [32–39]	35 [32–41]	33 [30–38]	37 [34–41]	0.051
pH 12 h after admission	7.33 [7.27–7.39]	7.31 [7.27–7.38]	7.35 [7.31–7.44]	7.29 [7.23–7.35]	0.016
${\text{HCO}}_{3}^{-}$ 12 h after admission, mEq/L	18.3 [15.5–19.9]	17.4 [15.0–19.5]	19.2 [17.1–21.4]	17.6 [15.1–19.3]	0.027
Lactate 12 h after admission, mEq/L	3.2 [2.3–5.4]	3.8 [2.5–6.7]	3.0 [2.2–4.3]	3.9 [2.5–5.9]	0.12
Mean blood pressure 12 h after admission, mmHg	82 [68–98]	81 [68–91]	87 [75–102]	79 [55–97]	0.088
PaO_2_ 24 h after admission, mmHg	145 [99–237]	80 [73–94]	135 [121–156]	297 [240–419]	<0.001
PaCO_2_ 24 h after admission, mmHg	36 [32–40]	37 [34–40]	34 [32–40]	38 [36–41]	0.096
pH 24 h after admission	7.36 [7.29–7.42]	7.35 [7.29–7.39]	7.40 [7.32–7.43]	7.32 [7.21–7.38]	0.004
${\text{HCO}}_{3}^{-}$ 24 h after admission, mEq/L	20.4 [17.5–21.9]	19.2 [16.9–20.9]	21.3 [20.0–22.3]	18.6 [15.7–22.2]	0.011
Lactate 24 h after admission, mEq/L	2.2 [1.4–3.8]	2.7 [1.4–3.7]	1.7 [1.2–2.5]	3.5 [2.1–5.7]	0.001
Mean blood pressure 24 h after admission, mmHg	79 [64–91]	78 [63–89]	82 [73–94]	71 [56–89]	0.044
30-day survival	52 (47.3)	13 (44.8)	27 (58.7)	12 (34.3)	0.096
30-day favorable neurological outcome	14 (12.7)	2 (6.9)	8 (17.4)	4 (11.4)	0.41

*Continuous variables are presented as median [interquartile range]. Categorical variables are presented as count (percentage)*.

${\text{HCO}}_{3}^{-}$, *bicarbonate ion; PaCO_2_, partial pressure of arterial carbon dioxide; PaO_2_, partial pressure of arterial oxygen; ECMO, extracorporeal membrane oxygenation*.

### Logistic Regression Analysis

The results of the multivariate logistic regression analyses for 30-day survival and 30-day favorable neurological outcome are shown in Table 3. Mild hyperoxia was not significantly associated with survival, compared with normoxia as reference (adjusted OR, 1.06; 95% CI: 0.30–3.68; *p* = 0.93). Furthermore, severe hyperoxia was not significantly associated with survival, compared to normoxia (adjusted OR, 1.05; 95% CI: 0.27–4.12; *p* = 0.94).

**Table 3:** Multivariate logistic regression analyses of 30-day survival and 30-day favorable neurological outcome after cardiac arrest.

	**Adjusted OR (95% CI)**	***p*-value**
30-day survival		
Normoxia	Reference	
Mild hyperoxia	1.06 (0.30–3.68)	0.93
Severe hyperoxia	1.05 (0.27–4.12)	0.94
30-day favorable neurological outcome		
Normoxia	Reference	
Mild hyperoxia	1.66 (0.26–10.65)	0.60
Severe hyperoxia	1.12 (0.15–8.39)	0.91

*A multiple propensity score was calculated after adjusting for the following factors: age, sex, type of witness, bystander administration of CPR, initial cardiac rhythm, pre-hospital shock delivery, pre-hospital adrenaline administration, etiology, percutaneous coronary intervention, intra-aortic balloon pump use, and time from arrest to ECMO pump-on. Adjusted ORs were calculated controlling for the multiple propensity score and variables at 24 h after admission, including PaCO_2_, pH, HCO^3−^, lactate level, and mean blood pressure*.

*CI, confidence interval; OR, odds ratio*.

Mild and severe hyperoxia were not significantly associated with 30-day favorable neurological outcome, compared with normoxia as reference (adjusted OR, 1.66; 95% CI: 0.26–10.65; *p* = 0.60; adjusted OR, 1.12; 95% CI: 0.15–8.39; *p* = 0.91, respectively).

### Sensitivity Analysis

The results of the sensitivity analysis (that is, multivariate logistic regression analysis performed after grouping the patients by PaO_2_ level at 12 h after admission) were also similar. Mild and severe hyperoxia were not significantly associated with 30-day survival when compared with normoxia as reference (adjusted OR, 4.93; 95% CI: 0.61–39.9; *p* = 0.14; adjusted OR, 2.58; 95% CI: 0.32–20.8; *p* = 0.37, respectively).

## Discussion

### Main Findings

We investigated the relationship between hyperoxia and clinical outcomes in adult patients who underwent ECPR, focusing on PaO_2_ levels 24 h after admission. After adjusting for multiple confounders, both mild and severe hyperoxia were not significantly associated with 30-day survival and favorable neurological outcome.

### Effect of Hyperoxia in Patients With Cardiac Arrest

Hyperoxia increases the production of reactive oxygen species during reperfusion injury, leading to oxidative damage to mitochondrial respiration and cerebral energy metabolism. Oxidative modification of mitochondrial proteins can inactivate cerebral pyruvate dehydrogenase complex (17). Furthermore, oxidative stress activates the mitochondrial permeability transition pore, releasing NAD(H) into the cytoplasm and depleting cofactors essential for metabolism (18). This results in metabolic failure, which can lead to decreased brain glucose and oxygen consumption, increased lactate production, and delayed neuronal death (19).

Several observational studies and meta-analyses have shown that hyperoxia after cardiac arrest is associated with poor neurological outcomes and increased mortality (20–23). Therefore, current post-resuscitation guidelines recommend avoiding prolonged hyperoxia (8, 9).

### Effect of Hyperoxia in Patients Undergoing ECPR

Supraphysiological hyperoxia frequently occurs during veno-arterial ECMO, such as that administered during ECPR, depending on the fractional delivered oxygen concentration setting of the sweep gas (12). Therefore, cardiac arrest patients undergoing ECPR are at a high risk of hyperoxia.

Previous studies have reported an association between exposure to hyperoxia and poor clinical outcomes (mortality and impaired neurological status) in cardiac arrest patients who undergo ECPR (10–12). The results of those previous studies are not consistent with those of the present study. However, confounding factors were not adjusted or controlled for in the previous studies (10–12). In addition, the site from which blood samples for gas analysis were obtained in those studies is unclear (11, 12). Therefore, the PaO_2_ levels used in the studies may not have been representative of cerebral oxygenation.

### Clinical Applications and Strengths of the Present Study

In this study, we focused on the PaO_2_ levels measured during ECMO and found that avoiding hyperoxia may not improve the clinical outcomes of patients who undergo ECPR. These results may be useful in the administration of ECMO to cardiac arrest patients during ECPR. More important than oxygenation are the prognostic determinants in patients undergoing ECPR, and they should not be downplayed by overemphasis on hyperoxia.

Our study has several strengths. First, we used blood samples from the right upper limb for blood gas analysis, which allowed us to investigate the relationship between cerebral oxygenation and prognosis. Second, we evaluated the effect of hyperoxia on clinical outcomes after controlling for various confounders using multiple propensity scoring.

### Study Limitations

This study has several limitations. First, this was a single-center retrospective cohort study with a small sample size; therefore, the study was probably underpowered to detect significant differences. Second, we did not adjust for cardiac function as a possible confounder. Peripheral veno-arterial ECMO is usually performed during ECPR (24). The retrograde blood flow from the ECMO mixes with the antegrade blood flow from the patient's own heart, creating a watershed condition called the Harlequin syndrome (North-South syndrome) (25). Therefore, the cardiac function of the patient is an important factor. However, we were unable to collect data on cardiac function. Furthermore, in this study, we only analyzed blood gas data at 12 and 24 h after admission. Therefore, the effects of oxygenation in the shorter or longer term are unknown. Well-designed studies that will eliminate or minimize these limitations are therefore required in the future.

## Conclusions

In OHCA patients who underwent ECPR, no significant association was found between hyperoxia at 24 h after admission and 30-day survival. These results may be useful in the administration of ECMO to cardiac arrest patients during ECPR. However, a well-designed study is needed to overcome the specific problems associated with ECMO administration, including Harlequin syndrome.

## Data Availability Statement

The datasets presented in this article are not readily available because approval from the Ethics Committee of the study institution is necessary for accessing them. Requests to access the datasets should be directed to MaK, kashiura@me.com.

## Ethics Statement

The studies involving human participants were reviewed and approved by the Institutional Review Board of Tokyo Metropolitan Bokutoh Hospital. Written informed consent for participation was not required for this study in accordance with the national legislation and the institutional requirements.

## Author Contributions

MaK conceived this study. MiK and KS collected the data. MiK and MaK statistically analyzed the data. MiK, MaK, and HY interpreted the data. MiK drafted the manuscript. All the authors contributed substantially to the study design, approved the manuscript, and agree to be accountable for this work.

## Conflict of Interest

The authors declare that the research was conducted in the absence of any commercial or financial relationships that could be construed as a potential conflict of interest.

## Publisher's Note

All claims expressed in this article are solely those of the authors and do not necessarily represent those of their affiliated organizations, or those of the publisher, the editors and the reviewers. Any product that may be evaluated in this article, or claim that may be made by its manufacturer, is not guaranteed or endorsed by the publisher.

## Abbreviations

CI: confidence interval
CPR: cardiopulmonary resuscitation
ECMO: extracorporeal membrane oxygenation
ECPR: extracorporeal cardiopulmonary resuscitation
OHCA: out-of-hospital cardiac arrest
OR: odds ratio.
